# Rapid plasticity of visually evoked responses in rat monocular visual cortex

**DOI:** 10.1371/journal.pone.0184618

**Published:** 2017-09-14

**Authors:** Trevor C. Griffen, Melissa S. Haley, Alfredo Fontanini, Arianna Maffei

**Affiliations:** 1 Program in Neuroscience, Stony Brook University, Stony Brook, New York, United States of America; 2 SUNY Eye Institute, Syracuse, New York, United States of America; 3 Medical Scientist Training Program, Stony Brook University, Stony Brook, New York, United States of America; 4 Department of Neurobiology and Behavior, Stony Brook University, Stony Brook, New York, United States of America; University Medical Center Goettingen, GERMANY

## Abstract

Sensory cortical circuits are shaped by experience during sensitive periods in development. In the primary visual cortex (V1) altered visual experience results in changes in visual responsiveness of cortical neurons. The experience-dependent refinement of the circuit in V1 is thought to rely on competitive interactions between feedforward circuits driven by the two eyes. However, recent data have provided evidence for an additional role of cortico-cortical circuits in this process. Indeed, experience-dependent changes in intracortical circuits can be induced rapidly and may result in rapid-onset functional changes. Unilateral occlusion of vision rapidly alters visual responsiveness, synaptic strength and connectivity of local circuits in the binocular region of V1 (V1b), where the inputs from the two eyes converge. In the monocular region of rodent V1 (V1m), where feedforward inputs from the ipsilateral eye are virtually absent, visual deprivation induces rapid plasticity in local circuits; however, functional changes seem to occur only after long periods of deprivation. In V1m there is currently no evidence for functional changes occurring within a time window compatible with that of local circuit plasticity. Here, we probed the visual responsiveness of neurons in rat V1m and assessed the effect of one day unilateral eye lid suture on single neuron visual responses. We report a novel form of plasticity within V1m that occurs on a timescale consistent with the earliest known changes in synaptic strength. Our data provide new insights into how sensory experience can rapidly modulate neuronal responses, even in the absence of direct competition between feedforward thalamocortical inputs.

## Introduction

Visual deprivation alters the responsiveness of neurons in primary visual cortex (V1) [1, 2]. It has been proposed that the changes in visual responsiveness rely on competitive interactions between the inputs from the two eyes [2, 3]. In support of this idea, it was shown that in regions of V1 where inputs from the two eyes converge (binocular V1, V1b) [4] changes in responsiveness become detectable rapidly after the onset of the manipulation [5, 6]. Differently, in regions of V1 innervated only by direct inputs from the contralateral eye (monocular V1, V1m) [4] changes in responsiveness to visual stimuli require longer periods of deprivation [6]. Recently, it was shown that in V1b, reorganization of visual maps and changes in neural activity occur more rapidly than previously anticipated and it was proposed that these rapid changes are due to plasticity within intracortical circuits [7]. Intracortical circuits within V1m are also affected by visual experience as evinced by recordings from acute brain slices, where changes in neuronal excitability and synaptic strength of recurrent connections in the hemisphere contralateral to the closed eye become detectable rapidly after the onset of the visual manipulation. Together, these results suggest that altering visual experience could in principle induce rapid functional changes in the monocular region of V1, where feedforward inputs from the ipsilateral eye are virtually absent [4, 8, 9].

Here, we asked whether a unilateral 24 h visual deprivation is sufficient to alter visually evoked responses in V1m neurons. To do that we used brief flash stimuli presented independently to each eye in order to broadly recruit neurons in V1m. To quantify single neuron activity, we obtained whole cell recordings in V1m of anesthetized rats, and reconstructed their location and morphology. We then used retrograde labeling to identify neurons projecting to V1m. Once membrane potential and spiking responses evoked by the visual stimuli were determined, we asked whether 24 h of unilateral visual deprivation can modulate flash-evoked single neuron responses in V1m. We show that V1m neurons respond to independent stimulation of both eyes, though responses to the ipsilateral eye had a significantly delayed onset, consistent with a potential polysynaptic origin. Our tracing studies suggest that these responses may have a cortico-cortical origin. A 24 h visual deprivation potentiated the late-onset polysynaptic ipsilateral response, while leaving the short-latency contralateral response unaffected. The change in amplitude of the late-onset ipsilateral response led to a shift in the interocular bias of V1m neurons toward the open eye. Our results show that altered visual experience can rapidly alter visually evoked responses in V1m neurons and propose an additional mechanism for the functional effects of visual deprivation.

## Experimental procedures

### Subjects

Experimental procedures were approved by the Stony Brook University Institutional Animal Care and Use Committee and followed National Institute of Health guidelines. Recordings were obtained from 45 Long-Evans rats of both sexes on postnatal day 26–33. Animals had *ad libitum* access to chow and water and were housed on a 12 h light/12 h dark cycle.

### Visual deprivation

Monocular lid suture (24 ± 2 h) was performed as described on a subset of subjects (*n* = 15 rats) [10]. Briefly, animals were anesthetized with an intraperitoneal injection of (in mg/kg): 70 ketamine, 3.5 xylazine hydrochloride and 0.7 acepromazine maleate. Antibiotic ointment and artificial tears were applied to both eyes, and then one eye was closed shut with 4–5 mattress sutures. Rats were placed back in their home cage only after fully recovered.

### Surgery

24 h after the onset of visual deprivation, rats were anesthetized for recordings as described in [11]. Surgical levels of anesthesia were induced with 1.2 g/kg urethane and 40 mg/kg pentobarbital. Anesthesia was maintained with additional doses of urethane (10–20% of induction) as needed. Bupivacaine hydrochloride was injected at incision points to ensure analgesia. Body temperature was maintained at 37°C with a heating pad (FHC Inc.). Lid sutures were replaced under low light with 1–2 loose sutures for easy reopening prior to recording. Rats were then placed on a stereotactic apparatus (Narishige) and a craniotomy (~1 mm diameter) was made at 89% of the distance between bregma and lambda either 2.8 mm (V1m) or 4.2 mm (V1b) lateral to the midline either ipsilateral to or contralateral to the sutured eye. Immediately before recording, a durotomy was performed and lid sutures were removed. Care was taken to keep the room as dark as possible.

### Whole-cell recordings

Whole-cell recordings were obtained using standard procedures for blind patch clamp [12]. 4–8 MΩ borosilicate patch pipettes were filled with intracellular solution (in mM): 116 K-Gluconate, 10 KCl, 1 HEPES, 4 Mg-ATP, 0.3 Na-GTP, 10 Na-Phosphocreatine; ~7.35 pH; ~295 mOsm; biocytin 0.1%. Capacitance was compensated and series resistance was partly (10–20 MΩ) compensated online. Correction for liquid junction potential was not applied. To be included in the analysis recorded cells included had to have resting membrane potential below -50 mV (with less than 5 mV change during recordings), and series resistance below 100 MΩ (with less than 20% change during recording). Depth of recording for each cell was corrected based on the angle between the pipette and the surface of the brain as estimated from recovered pipette tracks: 15° for V1m and 28° for V1b. Recordings were obtained 380–1060 μm below the pia. The location of recorded neurons in V1m and V1b was confirmed by referencing our histological preparation to the rat brain atlas (Paxinos), and in reference to a number of previous studies in which the borders between V1b and V1m were revealed histologically and with transynaptic tracing from the eyes [4, 9, 13].

### Visual stimulation and data analysis

Data were acquired in current clamp mode at 20 kHz with a Digidata 1440A board using Clampex 10 (Molecular Devices). Visual stimuli were sets of ten 5 ms flashes delivered to one eye at 0.17 Hz. Analysis was performed on sets of 10–20 stimuli. A custom-made apparatus was used to deliver flash stimuli: a 5V TTL pulse (Master-8, A.M.P.I.) was passed through a 7000 mcd, 25 mA forward current, 3.6 V maximum supply, 30° viewing angle, white-light LED (Radio Shack, model #276–017) in series with a 100 Ω resistor. The LEDs could deliver ~10 mW of power at steady state. LEDs were positioned at ~1mm from each eye at an angle of ~20° from the midline.

### Tracing and histology

To retrogradely label inputs to the superficial layers of V1m, rats, aged P28 (*n* = 4), were anesthetized as for visual deprivation and the retrograde tracer, cholera toxin, subunit B, conjugated to Alexa Fluor 555 (CTB, Life Technologies), was injected into V1m (6.5 mm posterior to Bregma; 2.8 mm lateral to midline; 0.52 mm below the pia) using a nanoject pressure injector (Drummond, 250 nL). One week after injection, rats were anesthetized and perfused transcardially with 4% paraformaldehyde in PBS. Fixed brains were sliced using a vibratome (100 μm, Leica VT100), incubated with Hoescht 33342 for 15 min (1:2000, Life Technologies), and mounted with Fluoromount-G (Southern Biotech). Confocal imaging was used to reconstruct the location of retrogradely labeled neurons. Specificity of the injection site was verified by the localized presence of retrogradely labeled neurons in the monocular region of the lateral geniculate nucleus of the thalamus (LGN).

To reconstruct location and morphology of neurons recorded with whole cell patch clamp, at the end of each experiment rats were overdosed with surgical anesthetics, then transcardially perfused with 4% paraformaldehyde in PBS. Brains were post-fixed in 30% sucrose in PBS, then sectioned (100 μm) on a vibratome and stained with DAB. To allow for unambiguous *post hoc* morphological identification, no more than 2 neurons were recorded from each craniotomy.

### Cell classification and inclusion

**Fig 1:**
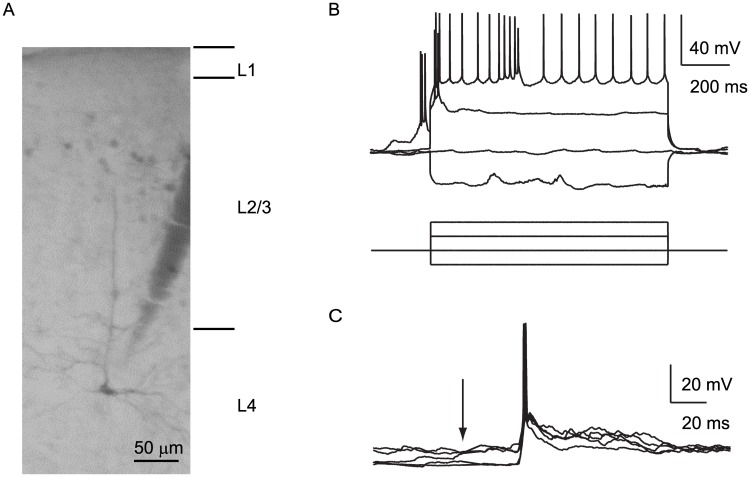
Whole-cell recordings of V1 neurons. (A) Recovered L4 V1m pyramidal neuron with representative morphology. The electrode track is visible next to the recovered cell. (B) Representative current injection/firing relationship of a V1 neuron in response to 1s sequential current injections (in pA: -250, 0, 250, 500). (C) Five representative, overlapped responses of a V1 neuron to stimulation of the contralateral eye. Arrow: stimulus onset.

Action potential threshold was measured as the point at which the derivative of the membrane potential reached 20 V/s [14]. Spike width was measured as the width at half-maximum spike height. Neurons with spike width > 0.5 ms, spike height > 30 mV and responding to current injection with bursts or regular spiking patterns were classified as excitatory [15]. Of cells fitting these criteria, 26 were recovered *post hoc*. 25 had typical pyramidal or star pyramidal morphology and were located in layers (L) 3, L4 and L5 (Fig 1A and 1B). One neuron had the morphology of a spiny stellate cell. 21 neurons could not be unambiguously identified *post hoc* but fit the inclusion criteria for excitatory neurons. Five neurons were classified as fast-spiking interneurons based on spike width (< 0.5 ms), spiking pattern in response to current injections, and multipolar morphology lacking dendritic spines. These neurons responded to light flashes, though due to the small sample size they were excluded from further analysis.

### Analysis and statistics

Analysis was performed with SigmaPlot (Systat Software, Inc.), Igor Pro (WaveMetrics), Excel (Microsoft) and Matlab (Mathworks). To analyze synaptic responses action potentials were removed with a 5 ms median filter. The peak velocity of the post-stimulus response was obtained by calculating the absolute value of the first derivative of the membrane potential and passing it through a 5 ms median filter. Individual derivative traces were excluded if distortions from an action potential burst remained. The response latency was quantified as the time from stimulus onset to the peak of the derivative during the post-stimulus response.

Action potentials evoked by visual stimuli were normalized using the area under the receiver operator curve (auROC) [16]. auROC values were obtained by comparing ten 100 ms baseline bins immediately preceding the stimulus to 100 ms post-response bins beginning at the peak of the membrane potential response. auROC values range from 0 to 1, with 0.5 as baseline and values higher or lower indicating increases and decreases in firing rate, respectively. The auROC interocular bias index (auROC-IBI) was calculated as *R*_contralateral_−*R*_ipsilateral_.

## Results

We used whole-cell recordings from L3, L4 and L5 neurons in acutely anesthetized rats to probe visually evoked changes in firing as well as membrane potential in response to brief flash stimuli (5 ms) delivered independently to the two eyes. The rapid change in luminance of a bright LED flash broadly activates cortical circuits, both in V1 and beyond [17], allowing us to record evoked responses originating thalamocortically and intracortically. The data presented here were obtained from a total of 43 V1m neurons (39 rats) and 4 V1b neurons (4 rats). During recordings neurons were filled with biocytin and recovered histologically to confirm their location within the anatomical region of V1 receiving binocular (V1b) or monocular (V1m) thalamic drive (Fig 1A) [10, 18–20].

### Delayed ipsilateral responses in V1m

Brief flash stimuli (5 ms) were delivered independently to each eye elicited responses in all recorded neurons (Fig 1). Amplitude and sign of visually evoked responses depended on the pre-stimulus membrane potential, with large depolarizing responses being detectable when neurons were in a hyperpolarized state and hyperpolarizing responses being evoked in neurons in a depolarized state at the time of stimulus delivery [21].

**Fig 2:**
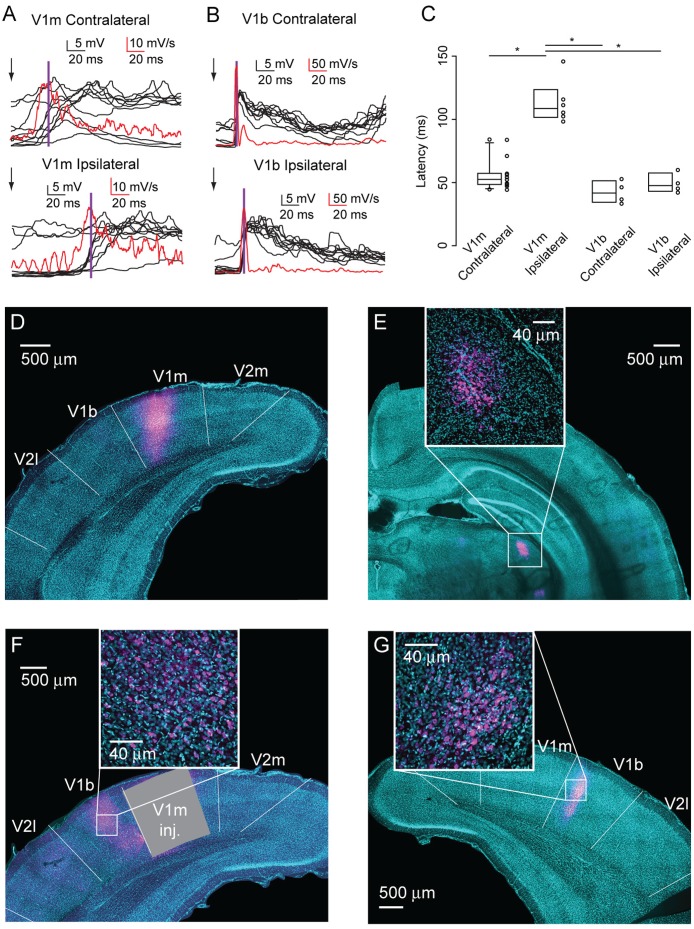
Delayed onset ipsilateral responses in V1m. (A) 10 overlapped membrane potential responses (black) to contralateral (top) and ipsilateral (bottom) eye stimuli recorded from a V1m neuron. The mean of the absolute value of the derivative of the action potentials (red) and the response latencies (purple) are superimposed. Arrow: stimulus onset. (B) Same as A, except traces are from a V1b neuron. (C) Response latencies of flash-evoked responses recorded in V1m and V1b. *p < 0.05, ANOVA on Ranks and *post hoc* pairwise multiple comparisons by Dunn’s Method. (D) Injection site of cholera toxin B in V1m. (E) Retrograde labeling specifically in the monocular region of the LGN confirms that the site of injection shown in D is in V1m. (F) Retrogradely labeled neurons in V1b of the injected hemisphere. The site of injection has been blanked to selectively show V1b labeled neurons. (G) Retrogradely labeled neurons in V1b of the hemisphere opposite to the injection site. For all images, insets show an enlarged portion of region of interest. Magenta: cholera toxin B; cyan: Hoechst. The boundaries between V1m and V1b were reconstructed based on the rat brain atlas [23].

All neurons recorded in V1m and V1b showed visually identifiable changes in membrane potential that were time locked to the stimulus and occurred within 200 ms of stimulus onset (V1m: Figs 1C and 2A; V1b: Fig 2B). Within the 200 ms time window from the stimulus both V1m and V1b neurons showed reliable responses to stimulation of either eye. The presence of ipsilateral eye responses in V1m may not be entirely surprising, given previous reports of flash-evoked responses recorded from both somatosensory and frontal cortices that suggest a broad recruitment of cortical circuits by flash stimuli [17, 22]. The magnitude and sign of individual evoked membrane potential deflections was dependent on the neurons pre-stimulus membrane potential, as shown for superficial neurons in the barrel cortex of behaving mice [21]. For single sweeps, if the neuron’s pre-stimulus membrane potential was depolarized close to action potential threshold at the time of the flash onset the evoked response was a negative deflection of membrane potential and stimulus-locked temporary reduction in spontaneous firing. However, for the same neuron, if the pre-stimulus membrane potential was hyperpolarized compared to action potential threshold, the flash evoked positive membrane potential deflections and evoked action potentials at the peak of the membrane deflection.

To verify that visually evoked responses in V1m and V1b were time locked with the flash stimulus, we measured the latency from stimulus onset to the peak rate of change of the membrane potential from baseline to the stimulus-locked evoked-response (Fig 2A and 2B). For all neurons, the peak rate of change corresponded to sharp transition in membrane potential time-locked to the flash stimulus. Thus, this method allowed us to determine membrane potential deflections driven by the stimulus across trials, independently of mean response amplitude or sign of individual responses.

V1m has been regarded as responding only to stimulation of the contralateral eye. However, our data show that flash stimuli to the ipsilateral eye evoked reliable responses in V1m neurons. This finding is not without precedent, as subthreshold binocular responses have been observed in neurons that show spiking responses to only one eye in the cat [24].

We compared the properties or ipsilaterally-evoked responses in V1m to those of responses evoked by contralateral eye stimulation in V1m, as well as to contralateral and ipsilateral responses recorded from neurons in V1b in naïve rats to assess possible differences. The latencies of ipsilaterally evoked responses in V1m (IER-V1m) were significantly longer than contralateral evoked responses in V1m (CER-V1m) and of ipsilateral and contralateral responses in V1b (IER-V1b; CER-V1b; Fig 2C; CER-V1m, 25–75%: 48.6–57.1 ms, median: 52.5 ms, *n* = 11 neurons (10 rats); IER-V1m, 25–75%: 102.6–116.0 ms, median: 108.6 ms, *n* = 6 neurons (5 rats); CER-V1b, 25–75%: 35.2–49.8 ms, median: 41.7 ms, *n* = 4 neurons (4 rats); IER-V1b, 25–75%: 43.9–54.9 ms, median: 47.6 ms, *n* = 4 neurons (4 rats); *p* < 0.05, ANOVA on Ranks and *post hoc* pairwise multiple comparisons by Dunn’s Method).

The longer latencies of IER-V1m suggest that they likely have a polysynaptic origin. To identify possible sources of ipsilateral inputs to V1m we used retrograde tracing with cholera toxin B. Unilateral focal injections of tracers were localized to V1m using a Nanoject pressure injector (Fig 2D). The identity of V1m was confirmed by the selective retrograde labeling of the monocular region of the LGN (Fig 2E) [25]. Retrogradely labeled somata were also detected in V1b of both hemispheres (Fig 2F and 2G), supporting the interpretation that ipsilateral responses in V1m likely originate intracortically, within and across hemispheres. Together, our data indicate that neurons V1m can respond to stimulation of both eyes. The contralateral response has a fast-onset consistent with a thalamocortical site of origin, while the newly identified ipsilateral responses in V1m have a long latency that is consistent with cortico-cortical/callosal inputs. The presence of cortico-cortical and callosal inputs is confirmed by the retrograde labeling of neurons projecting to the area of V1m that was targeted for the electrophysiological recordings.

### Rapid experience-dependent changes in visual responsiveness in V1m

The convergence of fast-onset contralateral inputs and slow-onset ipsilateral inputs from V1b of both hemispheres suggests that manipulation of visual drive may affect the interocular bias of V1m excitatory neurons. To investigate rapidly-induced functional changes in visual responses, we compared the interocular bias of V1m neurons following a 24 h unilateral visual deprivation (*n* = 15 neurons from 15 rats). Responses to stimuli were normalized using the area under the receiver operator curve (auROC) method [16] and an interocular bias index (IBI) was quantified. Using the auROC method of spike rate normalization allowed us to compare stimulus induced changes in firing rate from neurons with widely varying baseline firing rates, including those whose firing rate during the baseline approached 0 Hz, and allowed us to accept all neurons regardless of the nature of their baseline firing or spiking responses.

First, we assessed that there were no qualitative or statistically significant differences in auROC-IBI of contralateral eye responses or ipsilateral eye responses between V1m neurons recorded in naïve animals and neurons recorded from the hemisphere ipsilateral to the open eye in visually deprived animals (Control; Wilcoxon rank-sum tests, *p* values ≥ 0.2). Given the similarity of the responses, these neurons were pooled into a single Control group.

We also assessed that Control neurons and neurons recorded from the hemisphere contralateral to the closed eye (Deprived), were recorded at comparable depths (Distribution of depths of recordings: Control, 25–75%: 470–690 μm, median: 540 μm, *n* = 28 neurons (24 rats); Deprived, 25–75%: 500–590 μm, median: 510 μm, *n* = 15 neurons (15 rats); Kolmogorov-Smirnov test, *p* = 0.58), and did not show significant differences in series resistance, action potential half-width, action potential threshold, resting membrane potential or baseline firing rate (Table 1). All neurons listed in Table 1 met the criteria for inclusion in the analysis described in the Methods. A few of the parameters we measured required repeated sampling, and were therefore quantified on subsets of the neurons in Table 1 whose recording duration was sufficient to allow repeated measurement for statistical analysis. The sampling size for each parameter is indicated.

**Table 1:** Biophysical properties of V1m neurons.

	Control	Deprived	*p* =
***n* =**	28 (24 rats)	15 (15 rats)	
**Series Resistance (MΩ)**	47.5–64.5, 52.5	49.5–60.9, 52.1	0.91
**Action Potential Threshold (mV)**	-38.6–-33.5, -36.6	-37.1–-32.0, -33.7	0.12
**Action Potential Half-Width (ms)**	0.91–1.13, 1.02	0.86–1.05, 0.93	0.33
**Resting Membrane Potential (mV)**	-69.9 –-59.6, 63.7	-69.3 –-58.9, -61.9	0.71
**Baseline Firing Rate (Hz)**	0.2–2.8, 1.3	1.0–1.8, 1.3	0.69

Statistical significance was assessed with Wilcoxon rank-sum tests. 25–75%, median.

**Fig 3:**
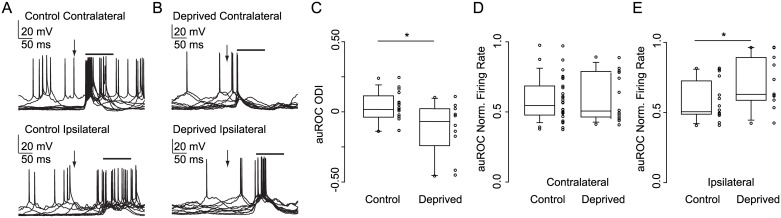
Rapid interocular bias plasticity in V1m. (A) Ten overlapped responses to contralateral (top) and ipsilateral (bottom) eye stimuli from a representative Control V1m neuron (recorded from V1m of the hemisphere contralateral to the open eye). (B) Ten overlapped responses to stimulation of the contralateral (top) and ipsilateral (bottom) eye of the Deprived hemisphere (recorded from V1m of the hemisphere contralateral to the closed eye). Note the large ipsilateral response. For (A) and (B), arrow: stimulus onset. Top bar: post-response bin. (C) auROC-IBI for V1m neurons recorded in the Control and Deprived hemispheres. *p = 0.037, Wilcoxon rank-sum test. (D) Responses to contralateral eye stimuli for V1m neurons from Control and Deprived hemispheres. *p* = 0.86, Wilcoxon rank-sum test. (E) Responses to ipsilateral eye stimuli for V1m neurons recorded in Control and Deprived hemispheres. *p = 0.041, Wilcoxon rank-sum test.

We then tested whether visual deprivation affected the responsiveness of V1m excitatory neurons to the contralateral versus ipsilateral stimuli by quantifying the auROC-IBI. This index ranges between 1 (maximal contralateral bias) and -1 (maximal ipsilateral bias). A 24 h visual deprivation shifted the interocular bias of V1m Deprived neurons (recorded from V1m contralateral to the closed eye) towards the open eye (Fig 3C; Control, 25–75%: -0.029–0.103 IBI, median: 0.023 IBI, *n* = 16 neurons (13 rats); Deprived, 25–75%: -0.174–0.023 IBI, median: -0.114 IBI, *n* = 10 neurons (10 rats); *p* = 0.037, Wilcoxon rank-sum test).

To determine whether this shift in interocular bias resulted from increased responsiveness to the open eye, decreased responsiveness to the closed eye, or changes in responsiveness to both eyes, we compared contralateral and ipsilateral eye responses of Control and Deprived neurons. Consistent with previous reports [6], brief visual deprivation did not affect contralateral responses of V1m excitatory neurons in the Deprived hemisphere (Fig 3D; Control, 25–75%: 0.479–0.678 auROC, median: 0.541 auROC, *n* = 28 (24 rats); Deprived, 25–75%: 0.463–0.782 auROC, median: 0.502, *n* = 15 neurons (15 rats); *p* = 0.86, Wilcoxon rank-sum test), although contralateral response latencies were slightly reduced (Control, 25–75%: 42.7–55.7 ms, median: 49.4 ms, *n* = 28 (24 rats); Deprived, 25–75%: 35.2–47.4 ms, median: 40.7 ms, *n* = 15 neurons (15 rats); Wilcoxon rank-sum test, *p* = 0.041).

Differently, V1m Deprived neurons showed potentiated responses to stimulation of the open (ipsilateral) eye (Fig 3E, Control, 25–75%: 0.490–0.693 auROC, median: 0.507 auROC, *n* = 16 neurons (13 rats); Deprived, 25–75%: 0.593–0.880 auROC, median: 0.631 auROC, *n* = 11 neurons (11 rats); Wilcoxon rank-sum test, *p* = 0.041). Response latencies for ipsilateral evoked responses in V1m were not affected (Control, 25–75%: 100.9–114.2 ms, median: 107.5 ms, *n* = 16 neurons (13 rats); Deprived, 25–75%: 95.4–119.8 ms, median: 100.2 ms, *n* = 11 neurons (11 rats); Wilcoxon rank-sum test, *p* = 0.16). Our data indicate that shifts in interocular bias in V1m can be induced even though the competitive interactions between thalamocortical afferents are absent. The events leading to the interocular bias shift in V1m differ significantly from what has been reported for V1b neurons, where interocular bias shifts depend on an initial decrease in contralateral (deprived) eye responsiveness followed by an ipsilateral (open eye) potentiation. In V1m, the early change in responsiveness depends on a potentiation of the long-delay, ipsilateral response evoked by flash stimulation of the non-deprived eye, while the responsiveness to stimulation of the deprived eye remains unaffected.

## Discussion

We have reported the presence of reliable, long-latency ipsilateral responses in V1m and unveiled two possible anatomical sources for these responses, recurrent and callosal inputs, using retrograde tracing. We have also shown that these ipsilateral responses are plastic. In V1m, brief (24 h) visual deprivation induced a novel form of plasticity of the interocular bias that relies on the potentiation of the newly identified long-latency ipsilateral responses that likely originate intracortically. The rapid potentiation of responsiveness to the non-deprived eye that we have observed is similar to the rapid potentiation of human visually evoked potentials elicited by stimulation of a non-deprived eye after a very brief monocular occlusion [26].

Ipsilateral responses in V1m differed from contralateral responses in V1m, as well as from ipsi- and contralateral responses in V1b, as their latency from stimulus onset was significantly longer, consistent with a polysynaptic origin. Retrograde labelling marked specifically the monocular domain of the LGN [25] indicating that our recording site is indeed in the monocular region of V1 [4]. The delay in visual evoked response and the retrograde labeling of the LGN suggest that it is highly unlikely that ipsilateral responses in V1m originate from direct thalamocortical projections from the ipsilateral eye [18, 19, 27]. The presence of retrogradely labeled neurons in V1b of both hemispheres supports the interpretation that the ipsilateral evoked responses in V1m depend on activation of polysynaptic intracortical circuits.

The ipsilateral visually evoked responses in V1m were exquisitely sensitive to changes in visual drive, as just 24 h of visual deprivation was sufficient to potentiate them, while it left the responses to flash-evoked contralateral responses unaffected. This set of changes results in a significant shift of the interocular bias in V1m. The interocular bias shift in V1m we report is induced more rapidly than the previously observed loss of responsiveness to the deprived eye in V1m [6]. The difference between our results and previous work in V1m [6] likely depends on the mode of stimulation. Ipsilateral patterned stimuli activate neurons according to their spatio-temporal preferences [6], while flash stimuli activate V1 broadly [17]. In addition, to identify the precise timing of membrane potential changes in response to stimuli we analyzed the rate of membrane potential changes. This approach allowed us to detect fast-onset and slow-onset changes in spiking within narrow time windows time locked to the individual neuron’s membrane potential response.

To date, interocular bias shifts as rapid as the one we report in V1m have only been observed in V1b outside L4, the primary recipient of thalamocortical afferents [7]. It has been proposed that such rapid changes may not rely on thalamocortical refinement, but depend on cortico-cortical interactions [7]. In V1b, a 24 h visual deprivation potentiated responses evoked by stimulation of both contralateral and ipsilateral eye in L2/3 of the hemisphere contralateral to the deprived eye [28]. The rapid increase in responsiveness of V1b was attributed to a weakening of inhibitory drive onto pyramidal neurons [28]. Differently, in V1m we observe a potentiation of the late-onset ipsilateral (open eye) response and no significant changes in contralateral (closed eye) response. If polysynaptic inputs from L2/3 of ipsilateral V1b contribute to the ipsilateral eye response in V1m that we have observed, it stands to reason that a rapid strengthening of these responses [28] could lead to a downstream potentiation of ipsilateral eye responses in V1m.

Competitive interactions between thalamocortical inputs to V1b are thought to drive the experience-dependent refinement of the circuit in V1b. It is generally thought that these competitive interactions are involved in the establishment of the contralateral bias, via stabilization of contralateral inputs. This hypothesis relies on data showing that in V1b the first significant change induced by visual deprivation is loss of responsiveness to the deprived eye that becomes significant several days after the onset of the visual manipulation, followed by a potentiation of open eye responses [5]. In V1m, contralateral (closed eye) responses were shown to decrease only following a much longer period of visual deprivation [6], and attributed to a non-competitive mechanism. In view of our findings, it is possible that certain ipsilateral stimuli that can activate V1m may favor competition between inputs from both eyes, and ultimately lead to a reduction in deprived eye responses in V1m.

Our data suggest that plasticity of visual responses occurs more rapidly than previously expected following visual deprivation within an area of cortex lacking significant ipsilateral thalamocortical drive. This finding is in agreement with data showing a prominent role for intracortical and callosal circuits in experience-dependent plasticity in V1m [29–31]. Differently from V1b, we show that a potentiation of open eye responses underlies the rapid interocular bias switch in V1m.

## Supporting information

S1 Data Tables reporting the analysis of electrophysiological recordings included in the figures. Worksheets contain data included in the plots.
(XLSX)
